# Hematocytological Clues of Peripheral Blood in Different Clinical Presentations of COVID-19

**DOI:** 10.30699/IJP.2023.561331.2963

**Published:** 2023-07-16

**Authors:** Seyede Fakhri Mohaghagh Zahed, Shahriar Dabiri, Abdolreza Javadi, Sajjadeh Movahednia, Manzumeh Shamsi Meymandi Pharm, Bahram Dabiri, Parisa Khorasani Esmaili, Mitra Sadat Rezaei, Mehrdad Faroukhnia

**Affiliations:** 1 *Pathology and Stem Cells Research Centers, Pathology Department, Afzalipour Medical School, * *Kerman Medical Sciences University, Kerman, Iran*; 2 *Department of Pathology, Shahid Beheshti University of Medical Sciences, Tehran, Iran*; 3 *Infectious Branch, Internal Medicine Department, Afzalipour Hospital, Kerman Medical Sciences University, Kerman, Iran*; 4 *Department of Pathology, NYU Long Island School of Medicine, NYU Langone Hospital, New York, United States of America*; 5 *Virology Research Center, National Research Institute of Tuberculosis and Lung Disease, Shahid Beheshti University of Medical Sciences, Tehran, Iran*

**Keywords:** COVID-19, Critical state, Cytogenesis, Cytological variants, Monocytes, Neutrophils, Peripheral blood smear

## Abstract

**Background:**

To gain insight into the pathogenesis and clinical course of COVID-19 from a historical perspective, we reviewed paraclinical diagnostic tools of this disease and prioritized the patients with a more severe form of disease admitted to intensive care units (ICUs). The objective was to better predict the course and severity of the disease by collecting more paraclinical data, specifically by examining the relationship between hematological findings and cytological variation of blood neutrophils and monocytes.

**Methods:**

This retrospective study was conducted on 112 patients with confirmed COVID-19 admitted to Imam Hossein Hospital (Tehran, Iran) from August to September 2020. Peripheral blood smears of these patients were differentiated according to several cytological variations of neutrophils and monocytes, and the correlation to the severity of the disease was specified.

**Results:**

The mean percentages of degenerated monocytes, degenerated granulocytes, and spiky biky neutrophils were significantly different among critical and non-critical patients (*P*<0.05). Degenerated monocytes and granulocytes were higher in critical patients as opposed to spiky biky neutrophils, which were higher among non-critical ones. Comparing the peripheral blood smears of COVID-19 patients (regarding pulmonary involvement in chest computed tomography [CT] scans [subtle, mild, moderate, and severe groups]), the twisted form of neutrophils was significantly higher in the subtle group than in the mild and moderate groups (*P*=0.003).

**Conclusion:**

Different cytological morphologies of neutrophils and monocytes, including degenerated monocytes, degenerated granulocytes, and spiky biky and twisted neutrophils, could help to predict the course and severity of the disease.

## Introduction

During the COVID-19 pandemic, we need more paraclinical data to predict the course and severity of the disease more easily. Although many studies have mentioned paraclinical factors that can predict the severity of the disease, there is still a lack of strong evidence linking certain hematological findings or procedures, such as bone marrow aspiration and biopsy, with the course and severity of this complex disease. 

COVID-19 patients are prone to hematological changes that manifest differently based on the severity of the disease (1-3). For example, lymphopenia is one of the most common findings in the peripheral blood smear of COVID-19 patients, which can also be a prognostic factor in the course of the disease. The ratio of neutrophils to lymphocytes and also platelets to lymphocytes could predict the course of the disease and its severity (1-4).

Moreover, leukocytosis with a predominance of neutrophils, relative lymphopenia, and monocytopenia can be the main hematological manifestations in COVID-19 patients. Also, white blood cells (WBCs), such as neutrophils, might show different morphological patterns in peripheral blood smears of these patients. Of these morphologic changes, more condensed nuclear chromatin, toxic granulation, and vacuolization of cytoplasm, as well as the C-shaped nucleus of neutrophils, have been mentioned (5).

Some other researchers have focused on the morphological changes of WBCs in peripheral blood smears of COVID-19 patients, such as neutrophils. They found that neutrophils seem to attack external pathogens (coronavirus) using neutrophil extracellular traps (NETs). This process in which nets of nuclear chromatin and protein with fiber bundles are formed is called NETosis (6). Other peripheral blood findings in COVID-19 patients include hyper-segmented neutrophils and red blood cell (RBC) changes (spherocyte, echinocytes, and rouleaux formation) (7).

Few studies have worked on bone marrow changes in patients and their correlation with clinical picture and prognosis. COVID-19 patients are prone to pancytopenia with decreased myeloid-to-erythroid ratio and normal cellularity according to their age (8).

Building on our previous work exploring the possible histogenesis of COVID-19 (9) and considering recent studies investigating specific morphologic findings of WBCs (particularly neutrophils and monocytes) in COVID-19 patients and their potential correlation with disease severity, we aimed to investigate these novel hematocytological findings to help clinicians better predict the course and severity of the disease.

## Material and Methods

This cross-sectional study was conducted on 112 patients who were admitted to Imam Hossein Hospital in Tehran, Iran, from August to September 2020. Patients included both confirmed cases and those with suspected infections. COVID-19 patients were diagnosed according to the World Health Organization (WHO) guidelines for the diagnosis of SARS-CoV-2 (10). The data of these patients were collected from the hospital information system of Imam Hossein Hospital, affiliated with Shahid Beheshti University of Medical Sciences, Tehran, Iran. We included patients who were at least 17 years old, diagnosed with COVID-19, and had no prior medical history of immune deficiency, thrombocytopenia, malignancy, or recent blood transfusion. 

COVID-19 was confirmed when the unique sequences of virus RNA were detected by real-time polymerase chain reaction (PCR). We also included patients diagnosed with possible COVID-19 disease according to the severity of symptoms and the imaging findings, even if their PCR was negative. Laboratory evaluations, including real-time PCR and complete blood count (CBC), were performed for all patients on the first day of admission. Chest computed tomography (CT) scans, using a 16-detector CT scan machine (Siemens Healthineers, Somatom, Germany), were performed (kvp: 100, mAs: 50-100, pitch: 1.5, and thickness: 4 mm). EDTA blood samples were taken using a 5-mL syringe for CBC analysis using a Siemens Advia 2120 hematology analyzer. Then, a peripheral blood smear was prepared for each patient and stained with the Wright-Giemsa dye to be evaluated and differentiated by the pathologist using a Primo Star Zeiss microscope (Carl Zeiss GmbH 07745, Jena, Germany). In this study, we differentiated the WBCs according to the specific morphological variants of neutrophils and monocytes that we assumed to have a relationship with the severity and course of the disease. 

**Fig. 1:**
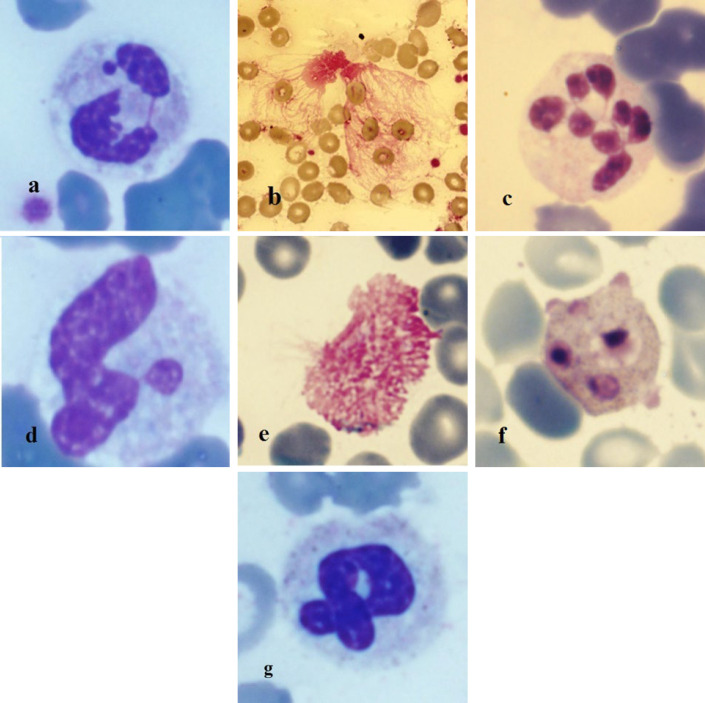
a) Spiky biky neutrophils, b) Degenerated neutrophils, c) Hypersegmented neutrophils, d) Drumstick neutrophils, e) Degenerated monocyte, f) Apoptotic cell, g) Twisted neutrophil

We named these specific variants as follows: a) spiky biky neutrophils: neutrophils with more than 1 spike, projecting from segments of neutrophil nuclei, b) degenerated neutrophils: neutrophils with released chromatin-based structures, almost like NETs (6), c) hypersegmented neutrophils: neutrophils that are defined as >5 lobes or ≥5% of neutrophils with 5 lobes (11), d) drumstick neutrophils: in this variant, there is a relatively small round detached piece of the male nucleus chromatin, e) degenerated monocytes: monocytes which are about to explode with an indistinct nucleus and irregular cytoplasmic border, f) apoptotic cells: WBCs with 1 or multiple pyknotic bits of chromatin and gray-pink cytoplasm, and g) twisted neutrophils: neutrophils with nucleus turning around its long axis (Figure 1).

Chest CT scans were reviewed by a radiologist, and the percentage of pulmonary involvement was scored and divided into 5 groups, including no involvement (<5%), subtle (5%-25% involvement), mild (26%-49% involvement), moderate (50%-75% involvement), and severe (>75% involvement) (12).

According to the severity of clinical symptoms, patients were classified into 2 groups of critical (admitted to the intensive care unit [ICU]) and non-critical (no need for ICU admission) patients.

The data were expressed as frequency and mean±SE and compared using the independent and paired *t* tests or 1-way analysis of variance (ANOVA), followed by the Tukey post-hoc test. The categorical values were analyzed using Fisher’s exact or chi-square tests. All analyses were performed using SPSS version 23 (SPSS Inc., Chicago, IL., USA). A 2-sided *P*<0.05 was considered statistically significant.

**Ethical Considerations**

The Local Research Ethics Committee of Shahid Beheshti University of Medical Sciences approved this study (code: IR.SBMU.RETECH.REC-.1400.349).

## Results

Of the 112 COVID-19 patients, 57 (50.9%) were women, and 55 (49.1%) were men with a mean±SE age of 58.6±1.6 years. In addition, 33 patients were in a critical state, while 79 patients were not. Two patients died because of COVID-19, and 110 were discharged. 

Regarding CBC parameters, the mean WBCs, neutrophils, lymphocytes, and monocytes absolute counts were 8.25×10^9^/L, 6.5×10^9^/L, 0.9×10^9^/L, and 0.4×10^9^/L, respectively. In addition, 30% of patients showed leukopenia, 21% had leukocytosis, and 66% were in the normal range regarding the WBC count. Also, 30% of the patients showed some degree of thrombocytopenia. Forty-three patients had >50% pulmonary involvement, and 36 patients had <50% pulmonary involvement, as shown in their chest CT scans.

The mean hemoglobin concentration during admission was significantly lower in critical patients (10.5) than in non-critical patients (11.5; *P*=0.04). The analysis of other CBC parameters and the clinical course of the disease, including critical status, degree of pulmonary involvement, and outcome, did not show any statistical significance. 

The mean percentage of degenerated monocytes, degenerated granulocytes, and spiky biky neutrophils were significantly different among critical and non-critical patients (*P*<0.05). Degenerated monocytes and granulocytes were higher in critical patients as opposed to spiky biky neutrophils, which were higher among non-critical ones (Table 1). 

The mean percentages of different cytological morphologies of neutrophils and monocytes according to the outcome are mentioned in Table 2.

The results showed that the group of patients who tested positive for SARS-CoV-2 by real-time PCR had a statistically significant increase in the mean percentage of degenerated monocytes compared to the group of patients who tested negative (*P*=0.02; Table 3).

**Table 1:** The mean percentage of different cytological morphologies of neutrophils and monocytes, according to sex and diagnosis

	Sex		P-value	Diagnosis		P-value
	**Male **	**Female**		**Critical **	**non-critical**	
Twisted neutrophil	7.9±0.5	7.8±0.5		7.2±0.6	8.1±0.4	
Degenerated monocyte	2.3±0.3	3.1±0.3		3.4±0.5	2.4±0.3	< 0.05
Degenerated granulocyte	3.1±0.4	4.3±0.5		4.7±0.5	3.3±0.4	< 0.05
Spiky biky neutrophil	21.8±1.1	18.0±1.1	< 0.05	16.2±1.3	21.4±0.9	0.002
Hyper-segmented neutrophil	4.8±0.7	3.9±0.6		3.5±0.5	4.7±0.6	
Drumstick neutrophil	3.2±0.4	4.8±0.5	< 0.05	4.2±0.7	4.0±0.4	
Apoptotic ell	0.4±0.1	0.6±0.1		0.7±0.2	0.5±0.1	

**Table 2:** The mean percentage of different cytological morphologies of neutrophils and monocytes according to the outcome

	Alive Expired
**Twisted neutrophils**	**7.8** ±0.3 ** 9.5** ±0.5
**Degenerated monocyte**	**2.7** ±0.2 ** 2.5** ±0.5
**Degenerated granulocyte**	**3.7** ±0.3 **2.5** ±0.5
**Spiky biky neutrophils**	**19.9** ±0.8 ** 17.0** ±9.0
**Hypersegmented neutrophils**	**4.3** ±0.4 ** 3.5** ±1.5
**Drumstick neutrophils**	**4.1** ±0.3 ** 3.5** ±1.5
Apoptotic cells	**0.5** ±0.07 ** 0.00**

**Table 3:** The mean percentage of different cytological morphologies of neutrophils and monocytes based on RT-PCR results

RT-PCR	Number	Mean
Twisted neutrophils detected Non-detected	89 14	7.5±0.3 9.0±0.9
Degenerated monocyte detected Non-detected	89 14	2.8±0.2 1.8±0.3
Degenerated granulocyte detected Non-detected	89 14	3.7±0.3 3.7±1.1
Spiky biky Neutrophils detected Non-detected	89 14	19.5±0.9 20.5±2.0
Hypersegmented neutrophils detected Non-detected	89 14	4.3±0.5 4.4±0.9
Drumstick neutrophils detected Non-detected	89 14	3.8±0.3 5.6±1.1

The mean percentage of twisted morphology of neutrophils was significantly higher in the subtle group than in the mild and moderate groups (10.5 vs. 7.7 and 7.2, respectively; P=0.02; Table 4).

**Fig. 2:**
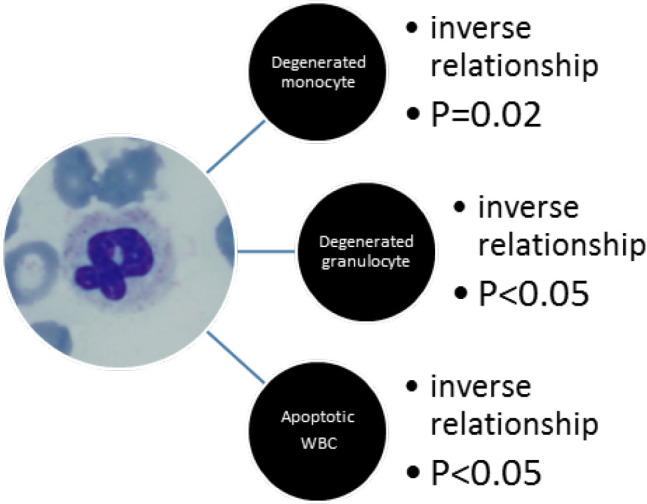
The correlation of the percentage of twisted morphology of neutrophils to other cytological morphologies of neutrophils and monocytes in the peripheral blood differential count

**Fig. 3:**
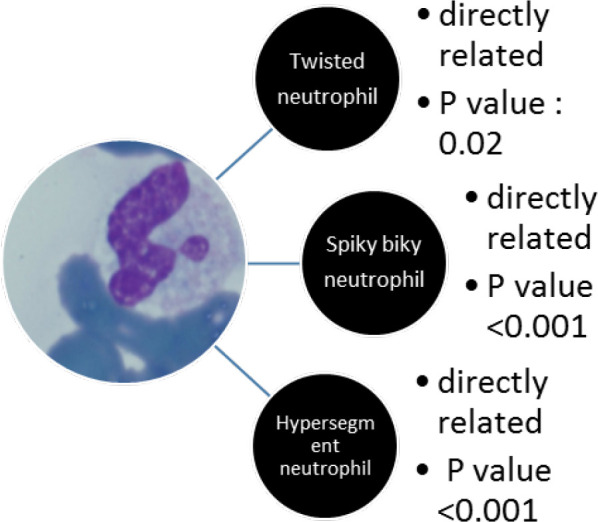
The correlation of the percentage of drumstick morphology of neutrophils to the rest of the cytological morphologies of neutrophils and monocytes in the peripheral blood differential count

The correlation of the percentage of the twisted and drumstick morphology of neutrophils to other cytological variants of neutrophils and monocytes in the peripheral blood differential counts are shown in Figures 2 and 3.

The percentage of degenerated granulocytes in the peripheral blood smear was directly related to the percentage of degenerated monocytes (P<0.001). Also, the percentage of hypersegmented neutrophils in the differential count was directly related to the percentage of spiky biky and twisted neutrophils (P<0.05 and P<0.001, respectively).

The percentage of spiky biky neutrophils was directly correlated with the hemoglobin level measured during admission (P=0.01). As previously mentioned, the spiky biky figure is related to milder symptoms and CT scan involvement, indicating that hemoglobin levels were higher in patients with a milder disease course.

The percentage of degenerated monocytes in differential blood count was associated with the severity of pulmonary involvement; in other words, patients with a higher percentage of degenerated monocytes tended to have more severe pulmonary involvement, as revealed by chest CT scans (*P*=0.01).

**Table 4:** The mean percentage of different

CT involvement	Number	Mean±SE
Twisted neutrophils Subtle Mild Moderate Severe	12 29 38 3	10.5±1.0 7.7±0.6 7.2±0.4 7.3±1.6
Degenerated monocyte Subtle Mild Moderate Severe	12 29 38 3	1.9±0.5 2.5±0.3 2.7±0.4 1.6±0.8
Degenerated granulocyte Subtle Mild Moderate Severe	12 29 38 3	3.4±0.9 4.4±0.6 3.5±0.5 1.0±0.6
Spiky biky Neutrophils Subtle Mild Moderate Severe	12 29 38 3	19.0±2.3 18.1±1.4 20.1±1.4 28.6±3.1
Hypersegmented neutrophils Subtle Mild Moderate Severe	12 29 38 3	6.1±1.5 3.7±0.8 4.1±4.5 7.6±5.3
Drumstick neutrophils Subtle Mild Moderate Severe	12 29 38 3	5.1±0.9 4.6±0.8 3.1±0.4 3.3±0.8
Apoptotic cells Subtle Mild Moderate Severe	12 29 38 3	0.4±0.2 0.4±0.1 0.6±0.1 0.0±0.0

## Discussion

Building on our previous work exploring the possible histogenesis of COVID-19, our current study investigated whether different cytological morphologies of neutrophils and monocytes in peripheral blood smears had any impact on the course of the disease, including critical status, the degree of pulmonary involvement, and outcome. 

Our findings suggest that degenerated monocytes, degenerated granulocytes, and apoptotic neutrophils were associated with more severe forms of the disease requiring hospitalization, as opposed to spiky biky, drumstick, and twisted variants of neutrophils which were more abundant in non- critical patients. Therefore, they could be a helpful tool to predict the course of the disease. 

COVID-19 patients are prone to hematological changes with different manifestations according to the severity of the disease (1-4). Liu and colleagues showed that lymphopenia was one of the most common findings in peripheral blood smears of COVID-19 patients, which can also be a prognostic factor in the course of the disease. They also mentioned that the increased ratio of neutrophils to lymphocytes and also a decrease in the hemoglobin concentration could be regarded as a risk of progression of the disease (1-13). Our study also revealed some degree of lymphopenia and lower level of hemoglobin during admission in patients with critical symptoms.

Hematological abnormalities, including lymphopenia, neutrophilia, and thrombocytopenia, were more pronounced in patients with severe disease compared with patients with non-severe forms of the disease (2,14,15,16)

Singh and coworkers also confirmed that leukocytosis with a predominance of neutrophils, relative lymphopenia, and monocytopenia could be the main hematological manifestation of COVID-19 patients. They also mentioned that WBCs (such as neutrophils) might show different morphological patterns in peripheral blood smears of COVID-19 patients. Of these morphological changes, more condensed nuclear chromatin, toxic granulation, vacuolization of cytoplasm, and C-shaped nucleus of neutrophils were mentioned (5).

In our study, we termed the NETosis-like variant of neutrophils as degenerated granulocytes, and it was significantly correlated with more chance of ICU admission. Our work will support the studies of Zhu *et al.*, Chiang *et al.*, and Yang *et al.* for NETs and NETosis works (6,17,18). NETosis also predicts COVID-19 severity and worse outcome (19).

Other peripheral blood findings in COVID-19 patients, including hypersegmented neutrophils and RBC changes (spherocyte, echinocytes, and rouleaux formation), have also been observed (7).

Zini and colleagues found other morphological WBCs changes in peripheral blood smears of patients with COVID-19, claiming that in the early stages of the disease, on day 1 of admission, some granulocytic abnormalities, including immature granulocytes, some degree of pseudo-Pelger-Huët morphology, and apoptotic cells, would be more evident, and after 1 week of starting anti-inflammatory and antiviral therapies, the most predominant changes would be in lymphocytes, which become activated in the course of the disease (20).

One study reported a leukoerythroblastic reaction with many smudge cells in the peripheral blood smear of a confirmed COVID-19 patient, stating that it could be a finding of COVID-19 infection; thus, it could help diagnose the disease more easily (21). Pezeshki and colleagues reported that the most frequent peripheral blood smear findings in COVID-19 patients were smudge cells, atypical lymphocytes, and giant platelets and schistocytes, but in this study, no relation was discovered between the progression of the disease and the morphological features mentioned above (22).

Nazarullah and coworkers also revealed that some morphological peripheral blood smear findings would be more pronounced in COVID-19 patients than in the control group, such as plasmacytoid lymphocytes, acquired Pelger-Huët anomaly, and left shift of granulocytic series (23).

Zhang and colleagues focused on monocyte number and morphologic and functional changes in COVID-19 patients using flow cytometry. They claimed that there was no difference in the number of monocytes between COVID-19 patients and the control group, but there were remarkable dissimilarities in their morphology and function, in a way that these changes were more pronounced in critical patients requiring ICU admission (24). 

Berber and coworkers showed that in severe cases of COVID-19, there was a higher number of band neutrophils and a lower count of segmented neutrophils, as in our study. They also claimed that a more severe disease course could be predicted by an increased pseudo–Pelger-Huët anomaly, mature lymphocyte ratio, decreased mature lymphocytes, and monocytes with vacuoles (25).

Although the expiring rate depended on the severity of illness and viral load, in critical patients, hemoglobin concentrations decreased, while neutrophils, WBC, and platelets count increased, especially it was seen in patients with more than 50% lung opacity involvement (26).

Another study also showed some morphological WBC anomalies in COVID-19 patients, including acquired neutrophilic nuclear projections (ANNP), acquired Pelger-Huet anomaly, shift to the left of myeloid series, plasmacytoid lymphocytes, toxic granulation, and vacuolization and apoptotic cells (27). Jafarzade and coworkers claimed that macrophages were the first line of defense against viral infections, especially in the respiratory tract (28). Therefore, this could explain why the percentage of degenerated monocytes in peripheral blood smears of patients was associated with a more severe form of the disease.

## Conclusion

Of the 112 patients evaluated for peripheral blood smear differentiation regarding the different cytological variants of neutrophils and monocytes, we noticed that the most common morphological variants were spiky biky and twisted neutrophils. Degenerated monocytes, degenerated granulocytes, and apoptotic neutrophils were associated with a more severe form of the disease requiring hospitalization, as opposed to spiky biky, drumstick, and twisted variants of neutrophils. Therefore, they could be used as a helpful tool to predict the course of the disease. However, further studies on larger patient populations with different strains of COVID-19 (such as Delta, Omicron, BA3, BA4, and potentially new strains in the future) are needed to confirm these findings.

## Funding

None.

## Conflict of Interest

None.
